# Annexin A2 in Virus Infection

**DOI:** 10.3389/fmicb.2018.02954

**Published:** 2018-12-05

**Authors:** Julia R. Taylor, Joseph G. Skeate, W. Martin Kast

**Affiliations:** ^1^Department of Molecular Microbiology and Immunology, University of Southern California, Los Angeles, CA, United States; ^2^Department of Obstetrics and Gynecology, University of Southern California, Los Angeles, CA, United States; ^3^Norris Comprehensive Cancer Center, University of Southern California, Los Angeles, CA, United States

**Keywords:** annexin A2, A2t, infection, virus, endocytosis, trafficking, virus lifecycle, epithelial cells

## Abstract

Viral life cycles consist of three main phases: (1) attachment and entry, (2) genome replication and expression, and (3) assembly, maturation, and egress. Each of these steps is intrinsically reliant on host cell factors and processes including cellular receptors, genetic replication machinery, endocytosis and exocytosis, and protein expression. Annexin A2 (AnxA2) is a membrane-associated protein with a wide range of intracellular functions and a recurrent host factor in a variety of viral infections. Spatially, AnxA2 is found in the nucleus and cytoplasm, vesicle-bound, and on the inner and outer leaflet of the plasma membrane. Structurally, AnxA2 exists as a monomer or in complex with S100A10 to form the AnxA2/S100A10 heterotetramer (A2t). Both AnxA2 and A2t have been implicated in a vast array of cellular functions such as endocytosis, exocytosis, membrane domain organization, and translational regulation through RNA binding. Accordingly, many discoveries have been made involving AnxA2 in viral pathogenesis, however, the reported work addressing AnxA2 in virology is highly compartmentalized. Therefore, the purpose of this mini review is to provide information regarding the role of AnxA2 in the lifecycle of multiple epithelial cell-targeting viruses to highlight recurrent themes, identify discrepancies, and reveal potential avenues for future research.

## Introduction

To successfully replicate, viruses must hijack and reprogram host cells to produce viral progeny. The life cycle of a virus consists of three main phases intimately reliant on host cell proteins and mechanisms. The first is cellular attachment and penetration. Attachment and penetration can occur through receptor-mediated endocytosis or through direct membrane fusion. Second, the viral genome is released for replication and protein expression. In this phase, the virus can rely on host enzymes to facilitate capsid uncoating or host machinery to replicate the viral genome. Finally, assembly and maturation yield newly constructed viral particles poised for release. In this stage, viral proteins can require post-translational modification by host factors, or intracellular transport systems for proper localization. During egress, virions are released by taking advantage of apoptosis, exocytosis, cell lysis, and by appropriating host membranes to bud directly from the cell.

This mini review aims to shed light on a host factor that has been repeatedly exploited for the benefit of viral infection: annexin A2 (AnxA2). AnxA2 is a membrane-associated protein implicated in a number of human, animal, and zoonotic infections. By addressing the involvement of AnxA2 in the context of multiple viruses and viral life cycle stages, we offer a broad perspective on an emerging host–pathogen interaction and highlight the complexities in AnxA2 biology.

### Annexin A2

Annexin A2 is a multifunctional calcium- and lipid-binding protein that is expressed in nearly all human tissues and cell types. AnxA2 exists as a monomer localized to the cytoplasm, vesicle-bound, or as a heterotetrameric complex termed A2t consisting of two AnxA2 monomers bridged by an S100A10 dimer found on the inner and outer leaflet of the plasma membrane. Both AnxA2 and A2t have been implicated in a wide range of intracellular processes including membrane domain organization, membrane fusion, vesicle aggregation, cytoskeletal-membrane dynamics, epithelial cell polarity, exocytosis, endocytosis, phagocytosis, and transcriptional regulation through binding of AnxA2 to RNA (reviewed in Gerke and Moss, 2002; Rescher, 2004; Bharadwaj et al., 2013; Hitchcock et al., 2014; Hajjar, 2015; Schloer et al., 2018).

More broadly, AnxA2 has been implicated in immune function, multiple human diseases, and viral infection (Hajjar, 2015; Tanida et al., 2015; Bećarević, 2016; Schloer et al., 2018). AnxA2 expression in some cancers can promote metastasis and function as a prognostic marker of recurrence and survival (Lokman et al., 2011; Zhang et al., 2013; Xu et al., 2015). This involvement of AnxA2 in human health and disease has prompted the development of pharmacological inhibitors of AnxA2 and A2t (Reddy et al., 2011, 2012, 2014; Liu et al., 2015), and these compounds are being explored in a growing number of therapeutic contexts. One class of inhibitors, for example, has been shown to block human papillomavirus (HPV) type 16 (HPV16) infection in cervical epithelial cells (Woodham et al., 2015). It is presumed that these A2t inhibitors disrupt the function of A2t during viral infection, though this specific mechanism still needs to be verified. Importantly, HPV is just one virus in a list of at least 13 viruses with known AnxA2 associations during binding, endocytosis, and egress (Table 1).

**Table 1:** Epithelial cell-targeting viruses that utilize Annexin A2.

Virus	Family	Envelope	Genome	Primary host tropism	Reference ∗ = addresses AnxA2 and heterotetrameric A2t
Human papillomavirus (HPV)	*Papillomaviridae*	−	dsDNA	Human	Woodham et al., 2012^∗^; Dziduszko and Ozbun, 2013^∗^; Taylor et al., 2018^∗^
Enterovirus 71 (EV71)	*Picornaviridae*	−	ssRNA	Human	Yang et al., 2011
Respiratory syncytial virus (RSV)	*Paramyxoviridae*	+	ssRNA	Human	Malhotra et al., 2003
Cytomegalovirus (CMV)	*Herpesviridae*	+	dsDNA	Human	Wright et al., 1994, 1995; Raynor et al., 1999^∗^; Derry et al., 2007^∗^
Hepatitis C virus (HCV)	*Flaviviridae*	+	ssRNA	Human	Lai et al., 2008; Backes et al., 2010^∗^; Saxena et al., 2012
Influenza A virus (IAV) H1N1	*Orthomyxoviridae*	+	ssRNA	Human	LeBouder et al., 2008^∗^
Avian influenza A virus (IAV) H5N1	*Orthomyxoviridae*	+	ssRNA	Avian/human	Ma et al., 2017
Measles virus (MV)	*Paramyxoviridae*	+	ssRNA	Human	Koga et al., 2018^∗^
Rabbit vesivirus (RaV)	*Caliciviridae*	−	ssRNA	Rabbit	González-Reyes et al., 2009
Infectious bronchitis virus (IBV)	*Coronaviridae*	+	ssRNA	Avian	Kwak et al., 2011
Classical swine fever virus (CSFV)	*Flaviviridae*	+	ssRNA	Porcine	Sheng et al., 2015; Yang et al., 2015
Bluetongue virus (BTV)	*Reoviridae*	−	dsRNA	Livestock	Beaton et al., 2002^∗^; Celma and Roy, 2011^∗^
Porcine reproductive and respiratory syndrome virus (PRRSV)	*Arteriviridae*	+	ssRNA	Porcine	Li et al., 2014; Chang et al., 2018

### Annexin A2–Virus Associations

**FIGURE 1:**
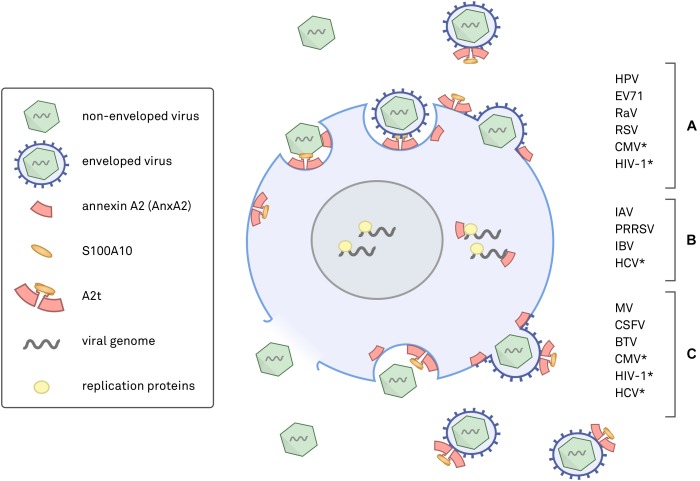
Schematic diagram of annexin A2 (AnxA2) and heterotetrameric AnxA2/S100A10 (A2t) in viral infections. Placements of AnxA2 depict where either AnxA2 or A2t have been implicated in a viral infection. **(A)** Viruses with evidence for AnxA2 involvement in attachment and entry, which can occur via endocytosis or by fusion of the viral envelope with the plasma membrane. **(B)** Viruses shown to utilize AnxA2 during genome replication and expression, a process that can occur in the cytoplasm or nucleus. **(C)** Viruses that have been shown to involve AnxA2 during assembly, maturation, and egress. Assembly can occur in the nucleus or in the cytoplasm, and release is achieved via cell lysis, apoptosis, exocytosis, or direct budding from the plasma membrane. ^∗^Evidence for AnxA2 involvement in more than one phase of the viral life cycle.

The annexin superfamily is highly conserved across eukaryotic phyla, from unicellular organisms to complex plants and animals (Moss and Morgan, 2004; Jami et al., 2012; Einarsson et al., 2016), and annexins have been associated with both human and non-human viral pathogens (summarized in Figure 1). This review focuses on seven viruses with direct links to AnxA2 during their lifecycle. To more confidently cross-compare cellular functions, we specifically discuss the viruses that target human epithelial cells. AnxA2 is utilized by HPV, enterovirus 71 (EV71), respiratory syncytial virus (RSV), and cytomegalovirus (CMV) during cell attachment and penetration (Wright et al., 1994, 1995; Raynor et al., 1999; Malhotra et al., 2003; Derry et al., 2007; Yang et al., 2011; Woodham et al., 2012, 2015; Dziduszko and Ozbun, 2013; Taylor et al., 2018), by hepatitis C virus (HCV) and influenza A virus (IAV) during replication (LeBouder et al., 2008; Backes et al., 2010; Saxena et al., 2012; Ma et al., 2017; Solbak et al., 2017), and by measles virus (MV) during assembly and maturation (Koga et al., 2018). In some cases, AnxA2 has been implicated in multiple life cycle steps of the aforementioned viruses, underscoring the importance of a more complete approach to understanding the role of AnxA2 in viral infection.

## Annexin A2 in Cell Attachment and Entry by Viruses

Virus attachment and entry into target cells occurs through host receptor-mediated endocytic mechanisms or less frequently, through direct fusion between virus envelope and the plasma membrane. The first steps of infection are attractive antiviral targets and are therefore studied extensively in a vast array of viral infections (selected reviews include: Mercer et al., 2010; Barrow et al., 2013; Yamauchi and Helenius, 2013; Helenius, 2018).

### Human Papillomavirus (HPV)

Persistent infection with HPV can lead to the development of a variety of anogenital and oropharyngeal cancers causing significant morbidity worldwide (Watson et al., 2008; Forman et al., 2012; Spence et al., 2016). HPV is a non-enveloped double stranded DNA (dsDNA) virus that enters basal keratinocytes through a non-canonical endocytic pathway while interacting with a number of host molecules (Spoden et al., 2008; Schelhaas et al., 2012; Raff et al., 2013; Day and Schelhaas, 2014; DiGiuseppe et al., 2017). In the search to identify an HPV uptake receptor, AnxA2 and A2t were discovered as central mediators of HPV entry and intracellular trafficking. Interestingly, it has been suggested AnxA2 and A2t have independent functions in HPV attachment and intracellular trafficking (Dziduszko and Ozbun, 2013). For example, it was shown that AnxA2 and A2t colocalize with HPV at the cell surface and that antibodies against AnxA2 alter entry kinetics (Dziduszko and Ozbun, 2013), but antibodies against the S100A10 subunit (Dziduszko and Ozbun, 2013) and targeted knock-out via CRISPR/Cas9 (Taylor et al., 2018) does not affect cellular entry *in vitro*. Furthermore, when the full A2t complex is knocked-out HPV infection is significantly reduced as measured by reporter gene transduction. However, when S100A10 alone is knocked-out, only a moderate reduction in infection is observed (Taylor et al., 2018), emphasizing the importance of delineating the roles of monomeric AnxA2 versus heterotetrameric A2t.

### Enterovirus 71 (EV71)

EV71 is a causative agent of hand, foot, and mouth disease (HFMD), a common infection in infants and children that can sometimes lead to severe illness and long term neurological conditions (Chan and AbuBakar, 2004). EV71 is a non-enveloped single-stranded RNA (ssRNA) virus that enters cells through an unknown dynamin-independent pathway (Yuan et al., 2018). In an effort to understand initial host–virus interactions, AnxA2 was identified as a cell surface attachment factor through anti-EV71 immunoprecipitation and mass spectrometric analysis of infected cells *in vitro* (Yang et al., 2011). Using immunofluorescence microscopy, these authors also demonstrated that AnxA2 and EV71 colocalize at the cell surface, and that pretreatment with recombinant AnxA2 (rAnxA2) or antibodies against AnxA2 yields reduced infectivity. Results from this work showed that AnxA2 and EV71 colocalize at the cell surface, but they did not address if the reduction in infectivity was due to reduced binding, entry, or replication. Furthermore, their yeast two-hybrid experiments showed that EV71 capsid protein VP1 interacted with the C-terminus of AnxA2, which may also implicate A2t as serving a functional role. Future studies investigating AnxA2 or A2t in EV71 endocytosis could yield interesting results given the varied implications of AnxA2 in this process.

### Respiratory Syncytial Virus (RSV)

Infants and elderly can develop severe lower respiratory disease from infection by RSV (Nair et al., 2010) – an enveloped ssRNA virus. The mechanism of RSV cellular entry is disputed, with independent reports suggesting plasma membrane fusion, clathrin-dependent endocytosis, and macropinocytic mechanisms (Kolokoltsov et al., 2007; Collins and Graham, 2008; Gutiérrez-Ortega et al., 2008; Krzyzaniak et al., 2013). Fucoidan is a polysaccharide that inhibits RSV infection *in vitro* and *in vivo.* Because it is assumed that fucoidan works by binding to RSV receptors, Malhotra et al. (2003) employed solid-phase-immobilized fucoidan as an affinity matrix to isolate potential RSV-binding partners on epithelial cells and identified AnxA2 using mass spectrometry. The authors show that treatment with rAnxA2 reduced RSV infection as measured by fluorescent focus assay 24h post-infection, but similar to Yang et al. they did not investigate the mechanism of infection reduction beyond cell surface interactions. An independent study did, however, demonstrate that AnxA2 is not involved in virus assembly (Shaikh et al., 2012). As was the case with EV71, a more detailed analysis of RSV endocytosis has the potential advance our understanding of AnxA2-mediated endocytosis.

### Cytomegalovirus (CMV)

Cytomegalovirus infection in immunocompromised individuals or through congenital transmission can lead to serious diseases including pneumonia and hearing loss (Fowler and Boppana, 2018). CMV is an enveloped dsDNA virus that is able to establish life-long persistence, and multiple CMV entry mechanisms have been described. Interestingly, it has been hypothesized that the viral entry route may actually influence the outcome of infection (Murray et al., 2018). Early work first discovered AnxA2 on the surface of CMV particles isolated from human fibroblasts, and found that rabbit antiserum against AnxA2 inhibited CMV infection *in vitro* (Wright et al., 1994, 1995). Using synthetic membrane systems and rAnxA2, Raynor et al. (1999) demonstrated enhanced binding of CMV if rAnxA2 was present and attributed fusion events to A2t. Follow up studies elucidated that AnxA2 is not essential for CMV entry (Pietropaolo and Compton, 1999; Esclatine et al., 2001), however, viral gene expression and completion of viral life cycle are dependent on AnxA2 and A2t (Derry et al., 2007) and progeny virions have been shown to contain both forms of AnxA2 on viral envelopes (Wright et al., 1994). These findings together suggest multiple roles of both AnxA2 and A2t for CMV trafficking and progeny egress.

## Annexin A2 in Virus Replication, Assembly, and Release

The ultimate goal of a virus is to produce and release progeny virions. In order to make new infectious particles, viruses must transcribe and replicate their genomes in either the cytoplasm or the nucleus of a host cell. To accomplish this, the virus orchestrates cellular factors to form replication complexes: organelle-like structures that form in the nucleus, the cytoplasm, endoplasmic reticulum (ER), or at the plasma membrane and shield cytoplasmic genome replication from host defenses (Den Boon et al., 2010; Schmid et al., 2014). Post-replication, virus particles must reassemble and traffic to the plasma membrane for release.

### Hepatitis C Virus (HCV)

Chronic infection with HCV can lead to the development of liver cirrhosis and hepatocellular carcinoma. HCV is an enveloped ssRNA virus that enters the host cell through endocytosis, replicates in ER-replication complexes, and exits via exocytosis (Farquhar et al., 2012; Lindenbach and Rice, 2014; Benedicto et al., 2015). Many viruses express non-structural (NS) proteins to aid in efficient and successful infection; accordingly, NS proteins often function within viral replication complexes. Lai et al. (2008) investigated host factors that might interact with a specific NS protein complex of HCV (NS3/NS4A) known to interact with actin filaments in kidney epithelial cells. NS3/NS4A expression and co-immunoprecipitation followed by mass spectrometry identified AnxA2 as an interacting host factor (Lai et al., 2008). Given that lipid rafts were demonstrated to be involved in the formations of HCV RC complexes and because AnxA2 is associated with both lipid rafts and interacted with NS4A (Gokhale et al., 2005), the authors published a follow-up study asking if AnxA2 aids in the formation of HCV replication complexes (Saxena et al., 2012). Their report details the localization of AnxA2 at HCV replication complexes via immunofluorescence and immuno-electron microscopy, a reduction in the number of these structures following AnxA2 siRNA silencing, and a reduction in HCV RNA synthesis. The reduction in RNA synthesis, however, was measured via HCV replicase activity although there was no observed change in relative mRNA levels. An independent report conclusively demonstrated that although monomeric AnxA2 colocalizes with HCV NS proteins, AnxA2 silencing has no direct effect on HCV RNA replication but causes a significant reduction in intra- and extracellular virus titers (Backes et al., 2010). Based on these findings, the authors conclude that AnxA2 is involved in viral assembly as opposed to replication. Interestingly, overexpression of AnxA2 led to an enrichment of HCV NS proteins at replication complex sites (Saxena et al., 2012), a mechanism that may in fact promote virus assembly and support the claim of these authors.

### Influenza A Virus (IAV)

Of the four types of influenza viruses, A, B, C, and D, influenza A viruses (IAV) and influenza B viruses cause epidemics of seasonal disease and respiratory infections. IAV type H1N1 and zoonotic avian IAV type H5N1 have both been associated with AnxA2 (LeBouder et al., 2008; Ma et al., 2017). IAV is an enveloped ssRNA virus that enters host cells via endocytosis, replicates in the nucleus, and buds from the plasma membrane for release (Salomon and Webster, 2009). It has been shown that AnxA2 and A2t are present on IAV H1N1 viral envelopes and that A2t, a plasminogen receptor, is responsible for the conversion of plasminogen to plasmin, a process involved in IAV replication (LeBouder et al., 2008). The authors demonstrated reduced viral titer after inhibiting plasminogen activation but did not tease out the precise involvement of AnxA2. An independent report investigated the role of AnxA2 in IAV H5N1 replication and found that silencing AnxA2 via siRNA inhibited viral protein expression and reduced progeny titer and proposed a mechanism by which AnxA2 bridged the gap between NS1 and p53, extending the amount of time cells could produce new virions (Ma et al., 2017). These data support the hypothesis that AnxA2 is involved in viral replication or assembly but do not preclude AnxA2 involvement during the preceding steps.

### Measles Virus (MV)

Measles is a highly contagious respiratory infection that is caused by MV – an enveloped ssRNA virus. After MV fuses with the host cell membrane, genome replication occurs in the cytoplasm and the virus is released by budding at the plasma membrane (Jiang et al., 2016). Knockdown of AnxA2 via shRNA in cervical epithelial cells (HeLa) caused reduced MV progeny virus generation 24 h post-infection, but did not affect MV entry and RNA replication (Koga et al., 2018). Normally, MV matrix protein (M protein) aids in connecting the viral capsid to the viral envelope and localizes to the plasma membrane where MV particles will form. Koga et al. (2018) went on to reveal that in the absence of AnxA2, M protein expression is decreased and mis-localized from the plasma membrane to the perinuclear space. Finally, the authors found that the observed M trafficking effect was due to monomeric AnxA2 versus A2t (Koga et al., 2018).

## Other Viruses and Species With AnxA2 Associations

In our mini review we have focused on epithelia-targeting viruses that cause disease in the human population and have all been shown to utilize AnxA2 or A2t in some capacity during their viral lifecycle. Outside of humans AnxA2, A2t, or a combination of the two have been implicated in the attachment and entry of rabbit vesivirus (RaV) (González-Reyes et al., 2009), the replication of porcine reproductive and respiratory syndrome virus (PRRSV; pig) and avian infectious bronchitis virus (IBV; chicken) (Kwak et al., 2011; Li et al., 2014; Chang et al., 2018), and in the assembly and release stages of classical swine fever virus (CSFV; pig) and bluetongue virus (BTV; livestock) (Beaton et al., 2002; Celma and Roy, 2011; Sheng et al., 2015; Yang et al., 2015). Additionally, there have been multiple reports of AnxA2-viral associations occurring within cell types beyond epithelial cells [e.g., Alphaherpesviruses with neuronal cells (Koyuncu et al., 2013) and human immunodeficiency virus with macrophages (Ma et al., 2004; Ryzhova et al., 2006; Rai et al., 2010; Woodham et al., 2016)], however, addressing viral tropisms for non-epithelial cells and how different cell types utilize AnxA2/A2t does not fall within the scope of this mini review.

Alas, a deeper understanding of how AnxA2 biology is manipulated during the course of viral infections may uncover novel treatment routes or expand our understanding of cellular biology in general.

Figure 1 summarizes where and how different viruses exploit AnxA2 and A2t and serves as a visual representation of the complexity of this subject. It should be noted that nearly half of these studies fail to address whether or not the observed AnxA2-associated effects are due to monomeric AnxA2 or heterotetrameric A2t (indicated in Table 1). Synthesizing our current understanding of AnxA2 in viral infections will reveal similarities between pathogens, highlight deficiencies in our experimental approaches, and help us better understand the diversity in AnxA2 functionality.

## Patterns and Potential

Multiple reports implicating AnxA2 in virus attachment and penetration base their conclusions on experiments that combine studying AnxA2-virus–membrane interactions with infection readouts post-AnxA2 manipulation. This strategy merely indicates that AnxA2 plays a role somewhere between cell attachment and life cycle completion. In-depth analysis of the virus life cycle in the context of AnxA2 could reveal novel information about AnxA2-mediated intracellular trafficking.

This mini review has provided a general overview of the diverse ways AnxA2 is utilized by viruses in epithelial infections in order to shed light on broad mechanistic patterns and identify potential avenues for future research. We conclude that AnxA2-mediated endocytosis may represent a distinct trafficking pathway utilized by multiple viruses. For example, EV71 and HPV are both suggested to travel through an undefined dynamin-independent pathway related to AnxA2 (Schelhaas et al., 2012; Yuan et al., 2018). The preferred entry route of RSV is also debated and similar to HPV, related to macropinocytosis (Kolokoltsov et al., 2007; Krzyzaniak et al., 2013; Mastrangelo and Hegele, 2013). The entry mechanisms of RSV, HPV, and CMV are proposed to be complex two-step processes involving proteoglycans and dependent on EGFR activation, HPV and RSV infection stimulate increased A2t translocation to the cell surface (Raff et al., 2013), a process which in itself is not well understood. Finally, both HPV and PRRSV infections involve vimentin – a protein that directly interacts with AnxA2. Together, these viruses may serve as models to study a unique AnxA2-dependent endocytic pathway.

Given the vast diversity in A2t functionality, it is probable that AnxA2 plays a highly complex and dynamic role in virus infection. As mentioned above, AnxA2 also has immunomodulatory effects (Woodham et al., 2014; Hajjar, 2015; Zhang et al., 2015), and it therefore possible that AnxA2 expression promotes a permissive environment for infection through modulation of innate immune responses. Additionally, AnxA2 may play a role in initiating adaptive immune responses against infections. For example, exogenous addition of A2t to the antigen-presenting cells of the epithelium, Langerhans cells (LC), induced suppression of immune activation and reduced Th-1 cytokine production *in vitro*, suggesting that A2t may function as an immune modulator in the epithelium. The small molecule inhibitor against A2t that was used in the HPV16 studies was also able to reverse HPV-induced immune suppression of LC populations, further supporting the notion that a deeper understanding of AnxA2 biology may reveal novel avenues for treatment options (Woodham et al., 2014).

Future studies should implement a holistic research approach that investigates the interactions between the host cell, the pathogen, and the immunological environment in which the viral lifecycle takes place. Ultimately, multi-modal research approaches may provide a more comprehensive understanding: we can learn more about AnxA2 endocytosis by studying different viruses, and we can use AnxA2 endocytosis as a model to better understand viral infection.

## Author Contributions

JT and WK conceptualized and designed the work. JS and WK contributed critical interpretation and revision for intellectual content. JT designed the figures.

## Conflict of Interest Statement

The authors declare that the research was conducted in the absence of any commercial or financial relationships that could be construed as a potential conflict of interest.
